# Brain Tumor Segmentation Based on Bendlet Transform and Improved Chan-Vese Model

**DOI:** 10.3390/e24091199

**Published:** 2022-08-27

**Authors:** Kexin Meng, Piercarlo Cattani, Francesco Villecco

**Affiliations:** 1 College of Information and Electrical Engineering, China Agricultural University, Beijing 100083, China; 2 Department of Computer, Control and Management Engineering, University of Rome “La Sapienza”, Via Ariosto 25, 00185 Roma, Italy; 3 Department of Industrial Engineering, University of Salerno, via Giovanni Paolo II 132, 84084 Fisciano, Italy

**Keywords:** Bendlet system, image expression, feature set, Shannon-cosine wavelet, segmentation

## Abstract

Automated segmentation of brain tumors is a difficult procedure due to the variability and blurred boundary of the lesions. In this study, we propose an automated model based on Bendlet transform and improved Chan-Vese (CV) model for brain tumor segmentation. Since the Bendlet system is based on the principle of sparse approximation, Bendlet transform is applied to describe the images and map images to the feature space and, thereby, first obtain the feature set. This can help in effectively exploring the mapping relationship between brain lesions and normal tissues, and achieving multi-scale and multi-directional registration. Secondly, the SSIM region detection method is proposed to preliminarily locate the tumor region from three aspects of brightness, structure, and contrast. Finally, the CV model is solved by the Hermite-Shannon-Cosine wavelet homotopy method, and the boundary of the tumor region is more accurately delineated by the wavelet transform coefficient. We randomly selected some cross-sectional images to verify the effectiveness of the proposed algorithm and compared with CV, Ostu, K-FCM, and region growing segmentation methods. The experimental results showed that the proposed algorithm had higher segmentation accuracy and better stability.

## 1. Introduction

With the rapid development of computed tomography (CT) and magnetic resonance (MR) imaging techniques, the role of cross-sectional imaging in the diagnosis of brain tumors has expanded. Cross-sectional images are capable of displaying diseased tissues and locations at high resolution with good contrast, thereby aiding in treatment planning. When working with cross-sectional images, one of the most complex problems is segmenting out some specific tissues. Segmentation helps doctors more accurately locate the lesion and assess the severity of the lesion, and is an essential and critical process in disease treatment [1]. Manual localization and segmentation of tumor regions by physicians is an expensive, time-consuming, and tedious task, and the segmentation results are not reproducible. Since cross-sectional images are low-contrast images, existing segmentation methods are often interfered with by bone and fat when detecting tumor contours. Clearly, brain tumor segmentation remains a perplexing task. 

Brain tumor segmentation methods mainly include segmentation methods based on supervised learning, semi-supervised segmentation methods, and unsupervised segmentation methods. Xu et al. [2] proposed a new joint motion feature learning architecture that can establish a direct correspondence between motion features and tissue properties, thereby determining the position, size and shape information of the infarction area of myocardial infarction. Zhao et al. [3] combined fully convolutional neural networks (FC-NNs) and Conditional Random Fields (CRFs) to segment brain tumor images slice-by-slice, and trained three segmentation models in axial, coronal and sagittal views to achieve better performance. Jiang et al. [4] proposed a novel dual-stream decoding CNN architecture that designs a separate branch to process edge-stream information, which can make the tumor edge clearer. Zheng et al. [5] proposed a four-dimensional (4D) deep learning model, based on three-dimensional convolution and convolutional long short-term memory (C-LSTM), which can more effectively learn the features of hepatocellular carcinoma (HCC) in multi-phase dynamic contrast enhanced (DCE) images. In medical image-based tumor segmentation, the main problem is insufficient labeled samples [6]. When segmenting tumor tissue in medical images, both supervised learning segmentation methods and semi-supervised segmentation methods suffer from insufficient labeled samples.

Unsupervised segmentation does not require ground-truth images as a criterion to train the model. Although there are several general segmentation methods, such as histogram thresholding [7], region growing [8], CV, and statistical clustering [9], etc., they have failed to achieve good results in the domain of brain tumor identification. Wavelet-based methods are widely used to solve difficult and hot problems, and their effectiveness has been proven in many applications, including data compression [10], signal processing [11], image enhancement [12], image compression [13], image segmentation [14], pattern recognition [15], etc. Wavelet analysis is able to refine and analyze complex information at multi-scales through scaling and translation operators. The commonly used two-dimensional wavelets are the tensor product of one-dimensional wavelets, and the number of directions is limited. The lack of directionality means wavelet transform is unable to make full use of the geometric regularity of the image, so it presents a step-like approximation to smooth contours or textures in an image. Owing to the above limitations, Curvelet [16] and Contourlet [17] were proposed and gradually applied. They increased the number of directions, while maintaining the advantages of two-dimensional wavelets. Raghunandan et al. [18] set a fixed window according to the sub-band relationship of the Contourlet, and used the SVM classifier to extract features in the wavelet domain of Contourlet for each window to achieve text recognition. Nayak et al. [19] presented a Pathological Brain diagnosis process by Curvelet sub-bands and entropy features in different scales and directions. Raikar et al. [20] utilized the multi-scale representation of Curvelet for rotator cuff disease diagnosis. In 2006, Guo et al. [21] introduced the shearing matrix and scaling matrix for geometric transformation into wavelet transform for the first time, and proposed the concept of Shearlet transform to realize multi-scale transformation with direction adaptation. Shearlet transform is widely applied, because it is capable of dealing with the anisotropic features of the image and capturing the geometric information of the edge. Sneha et al. [22] decomposed the image into main information and edge detail feature information through NSST to fuse multimodal medical images, which can help researchers study brain pathology more precisely. To accurately obtain the curvature information of the image, Amit et al. [23] proposed an improved adjustable non-subsampling Shearlet transform (ANSST) based on the Meyer window, but it still has great disadvantages for the extraction of edge curvature information. As we all know, contour curvature is one of the important features of the image, but the existing wavelet transforms lack curvature parameter. Lessig et al. [24] added curvature parameters into the Shearlet transform, resulting in a new wavelet transform “Bendlet”, which is the second-order Shearlet that can accurately express images and identify contour curvature features. Medical cross-sectional images are piecewise smooth images with obvious curvature characteristics, so Bendlet, based on multi-scale analysis, is more suitable for the analysis and processing of medical images.

Our main contributions can be summarized as follows:

(1) In this study, we propose a model that can detect the position of the tumor from a single image and delineate the tumor region by exploiting the similarity of the images themselves.
(2) The curvature of the left and right contours of a person’s brain tumor image is not the same, which affects the judgment of the location of the tumor. Existing registration algorithms are prone to wrongly mapping points in medical images. Therefore, we propose a registration algorithm based on Bendlet, which can obtain the curvature features of medical images and classify them to register and correct images.
(3) To solve the problem that the CV model cannot converge to the tumor position and the segmentation is incomplete, we propose the SSIM unit detection algorithm, which can roughly locate the tumor position from three aspects: brightness, structure, and contrast. Then, the CV model is improved by the Shannon-Cosine wavelet homotopy method, which further improves the segmentation accuracy.

The rest of the paper is organized as follows. Section 2 is our experimental methodology. The experimental results and discussion are presented in Section 3, while the conclusion appears in Section 4.

## 2. Materials and Methods

**Figure 1:**
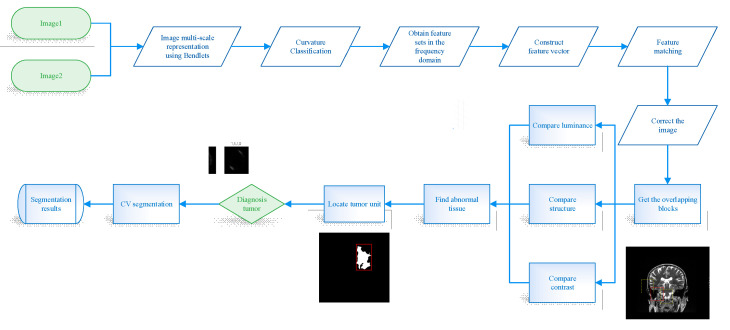
Schematic of the proposed brain tumors Segmentation method.

The proposed complete workflow for brain tumor detection is illustrated in Figure 1, where the proposed method uses the following basic steps: (1) Image registration using Bendlet transform, (2) Unit localization, and (3) Refined segmentation. Brain tumors are abnormal tissue growths in the brain that cause our brains to compress and deform, so we propose a multi-scale and multi-directional registration algorithm based on Bendlet. The Bendlet registration method is able to find enough feature points and establish the mapping relationship between the two images. Then, the cross-sectional images of the brain can be registered and corrected by non-rigid transformation.

### 2.1. Bendlet Registration Techniques

**Figure 2:**
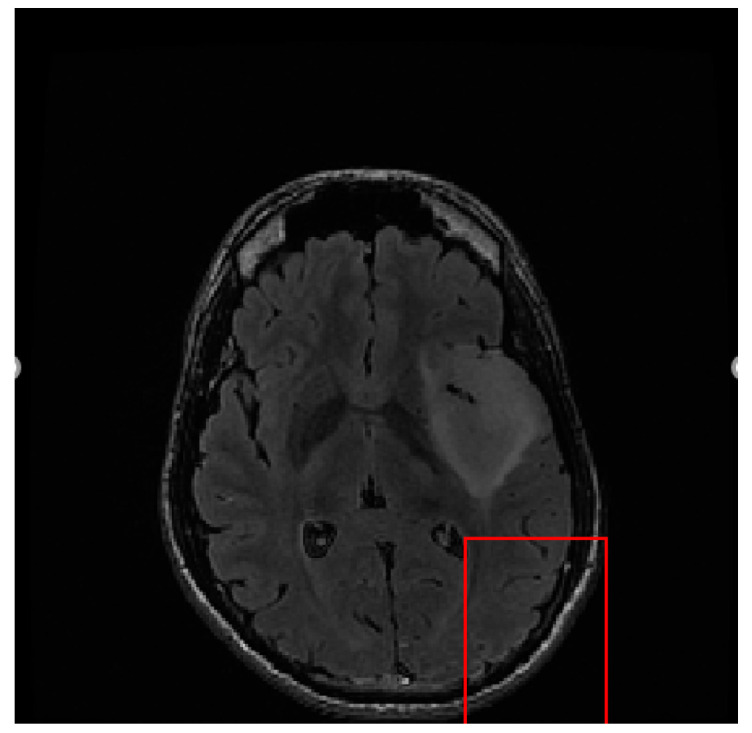
Tumor position detected in images without registration.

Our proposed tumor segmentation method is based on the asymmetry between the two brain hemispheres. However, the contour curvatures of the two hemispheres of the brain are not the same. If the tumor is detected directly without registration, it cannot be successfully detected, as shown in Figure 2.

#### 2.1.1. Bendlet System

The classical wavelet methods can detect singular points, have multi-resolution and localization characteristics, but cannot achieve the optimal expression of boundary curves. Shearlet introduced a shear matrix to control the direction, which is a multi-scale wavelet transform with good direction sensitivity and anisotropy. It is an extension of multi-dimensional, multi-scale, and multi-direction wavelet transform [25,26]. However, they also have disadvantages in characterizing and describing boundary curves, due to the lack of bending parameters. In medical imaging, boundary curves of tumors, bone and soft tissue segmentation curves provide valuable information, such as image structure. Curvature is an important parameter to characterize and describe these curves. Current orientation representation systems have achieved great success in extracting and characterizing boundary curves, but still fail to accurately classify curvature. 

Bendlet [27] introduced bending elements as another degree of freedom based on Shearlet to approximate piecewise smooth images, known as a second-order Shearlet transform. Compared with other wavelet transforms, Bendlet transform has great advantages in image approximation. The construction of Bendlet is different from the Shearlet in the scaling operator and shearing operator.

For *a* > 0 and *α* ∈ [0,1], the *a*− scaling is defined in Equation (1):

(1) $$A_{a,\alpha }:=\left(\begin{array}{cc} a & 0\\ 0 & a^{\alpha } \end{array}\right)$$

For *l* ∈ *N*, *r* = (*s*,*b*) and $r={\left(r_{1},\cdots ,r_{l}\right)}^{T}\in R^{l}$, the l−th order shearing operator is defined in Equation (2). The value *s* takes the role of shearing and *b* corresponds to a bending:

(2) $$S_{r}^{\left(l\right)}(x)=\left(\begin{array}{cc} 1 & \sum_{m=1}^{l}r_{m}x_{2}^{m-1}\\ 0 & 1 \end{array}\right){\left(x_{1},x_{2}\right)}^{T}$$

The shearing matrix is generated by letting *l* = 1, and the operator contains the characteristics of both shearing and bending by letting *l* = 2. The l−th order *α* Shearlet system is denoted as Equation (3):

(3) $$SH_{\psi }^{\left(l,\alpha \right)}(f)(a,r,t):=\langle f,\pi^{\left(l,\alpha \right)}(a,r,t)\psi \rangle$$

where $\psi_{a,r,t}(x)=a^{-\frac{1+\alpha }{2}}\psi \left(A_{a,\alpha }^{-1}S_{-r}^{\left(l\right)}(\cdot -t)\right)$. When *l* = 2, the above equation is considered as second-order Shearlet transform or Bendlet transform.

**Figure 3:**
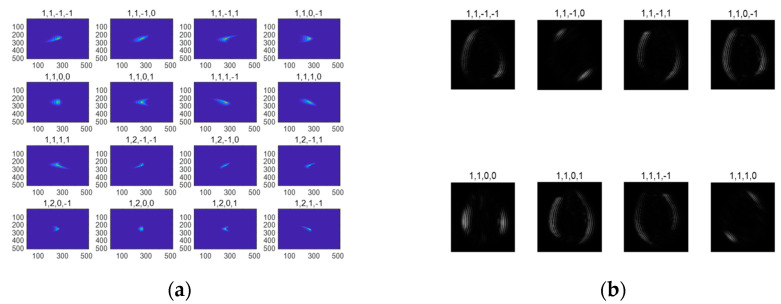
Bending elements of the Bendlets system and its representation of images. (**a**) Bending elements in spatial domain. (**b**) Multi-scale representation of contours in different directions by Bendlets system.

Figure 3a shows some bending elements of the Bendlet system in the spatial domain. The four parameters above the image are cone, scale, shear, bending. The Bendlet system has multi-scale characteristics ([1, 1, −1, −1], [1, 2, −1, −1]). The shear parameter controls different directions, and when the shear parameter is changed, the orientation of Bendlet elements change. Figure 3b shows the multi-scale representation of medical image contours in different directions by the Bendlet system. 

The decay rate of the Bendlet transform varies with *a*, *s*, *b* and *t*. Classification of curvatures can be completed by different decay rates. For a small boundary radius and large curvature, only the coefficient corresponding to the large bending elements decay slowly. As the radius increases, the curvature becomes smaller, and the coefficient decays the slowest for the small bending parameter. The decay rate is the lowest when the Bendlet element overlaps the boundary curve. Meanwhile, the curvature at the boundary can be obtained by matching the second-order Taylor expansion of the boundary with a circle with radius *r* > 0 and curvature, which can be calculated by Equation (4):

(4) $$K=\frac{2\left|b^{\prime }\right|}{{\left(1+{\left(s^{\prime }\right)}^{2}\right)}^{\frac{3}{2}}}$$

#### 2.1.2. Multi-Scale and Multi-Direction Registration Method Using Bendlet

To improve the level of medical diagnosis and treatment, it is necessary to analyze several images together to obtain comparative information about the patient. We compare and analyze normal cross-sectional images with brain lesions to recognize brain tumors. Tumors will squeeze normal brain tissue, causing flexible deformation. Note that any inaccuracy in registration or bias correction stages directly affects the precision of tumor segmentation. It is necessary to find enough corresponding feature points for flexible transformation to make the two images spatially consistent.

**Figure 4:**
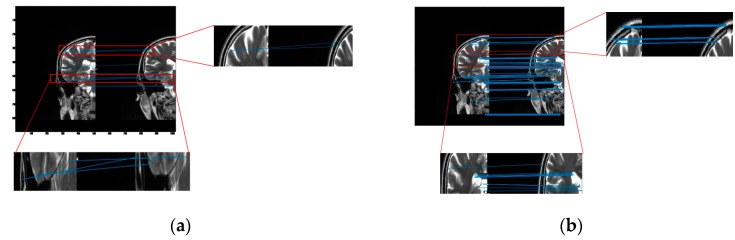
The Experimental results of image registration between two brain hemispheres (**a**) The registration result of SURF algorithm. When the SURF algorithm is applied to medical images, the feature points obtained are few and contain more misalignment points. (**b**) The registration result of the proposed method. Our method increases the number of registered points and reduces mis-matched points.

The cross-sectional images of the brain are piecewise smooth, and common registration algorithms are prone to false connection points (as shown in Figure 4a). As we can see from the images, the tissue usually does not follow any particular direction. Throughout the image, the tissue structures continually change direction, creating curved edges. For piecewise smooth medical images, the curvature becomes an important parameter to describe and characterize. Bendlet transform can achieve curvature classification by adding bending elements.

**Figure 5:**
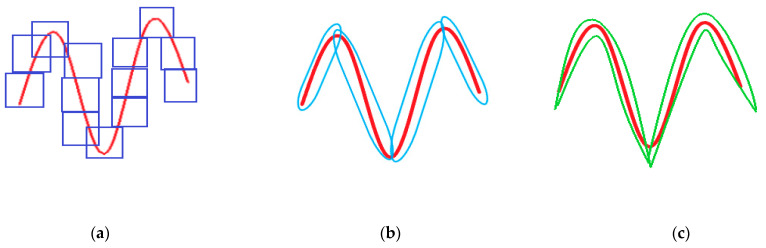
Capturing contour curve of cross-sectional images with wavelet, Shearlet, and Bendlet. (**a**) A huge amount of wavelet coefficients is required to detect contour curve information. (**b**) Few Shearlet coefficients are needed to complete detection. (**c**) Bendlet needs only 2 coefficients to fully detect curve information.

The traditional direction wavelet can detect the curvature through the coefficient response value, but it can only represent the information of three directions of the image, namely horizontal direction, vertical direction and diagonal direction. Since the shape of these elements is a square structure, a large number of coefficient responses are required to fully capture all the information, which also increases the noise inside the image. The Bendlet transform introduces bending parameters to enable bending characteristics. When approximating the curve, the energy can be concentrated on several coefficients. It requires fewer coefficients than ordinary wavelets to fully detect the curvature information in the image. As shown in Figure 5, To capture the curvature information, wavelet needs about 13 coefficient values, Shearlet needs 4 coefficients, and Bendlet needs only 2 coefficients to fully detect curved regions [28].

We transformed cross-sectional images of the brain to the frequency domain via Bendlet, analyzing the transformation coefficients at each scale. When the bending elements coincided with the curvature of the image, the coefficient response was very large, and contour information could be extracted from medical images by Bendlet. We introduced bending elements in Bendlet as registration elements to describe medical images at multi-scales and multi-directions. When the bending element was consistent with the curvature of the image, the point with large coefficient response was found as the registration point. Then, stable points at different scales were selected, and feature vectors were constructed to realize image registration and correction. As shown in Figure 4, our method had more connection points and no mismatch points.

### 2.2. The SSIM Region Detection

Brain tumors are the abnormal tissue structures on cross-sectional images relative to normal images. We can measure the similarity between normal images and brain tumor images in terms of brightness, structure, and contrast, and detect the region where abnormal tissue is located.

The luminance comparison is:

(5) $$l\left(x,y\right)=\frac{2\mu_{x}\mu_{y}+C_{1}}{{\mu^{2}}_{x}+{\mu^{2}}_{y}+C_{1}}$$

The contrast-based comparison is:

(6) $$c\left(x,y\right)=\frac{2\sigma_{x}\sigma_{y}+C_{2}}{{\sigma^{2}}_{x}+{\sigma^{2}}_{y}+C_{2}}$$

Comparison of the structure can be obtained through Equation (7):

(7) $$s\left(x,y\right)=\frac{\sigma_{xy}+C_{3}}{\sigma_{x}\sigma_{y}+C_{3}}$$

The three components are combined into a unique expression that is weighted with exponents *α*, *β* and *γ*:

*SSIM*(*x*,*y*)=[*l*(*x*,*y*)]^*α*^⋅[*c*(*x*,*y*)]^*β*^⋅[*s*(*x*,*y*)]^*γ*^ (8)

where, *u_x_* and *u_y_* are the mean of pixels in the image blocks *x* and *y*, σ*_x_* and σ*_y_* are the standard deviations of the pixels in the image blocks *x* and *y*, σ*_xy_* is the covariance between *x* and *y*, *C*_1_,*C*_2_,*C*_3_ are constants. The formulae: $\sigma_{xy}=\frac{1}{H*W-1}\sum_{i=1}^{H}\sum_{j=1}^{W}\left(X(i,j)-u_{x}\right)\left(Y(i,j)-u_{Y}\right)$, $u_{x}=\frac{1}{H*W}\sum_{i=1}^{H}\sum_{j=1}^{W}X(i,j)$, $\sigma_{x}={\left(\frac{1}{H*W-1}\sum_{i=1}^{H}\sum_{j=1}^{W}{\left(X(i,j)-u_{x}\right)}^{2}\right)}^{\frac{1}{2}}$. {*x*_1_,*x*_2_,*x*_3_,…,*x_n_*} show that the normal image is divided into *n* image blocks. The values {*y*_1_,*y*_2_,*y*_3_,…,*y_n_*} show that the brain tumor image is divided into *n* blocks. We can calculate SSIM values of every two image blocks using Equation (8), *SSIM* = {*s_1_*,*s_2_*,*s_3_*,…,*s_n_*}. The block with the smallest SSIM value is the image block where the brain tumor is located.

Segmenting within the unit area can reduce the interference of other organs and improve the accuracy of segmentation. First, we set the degree of overlap and the sliding window to split the image into overlapping blocks. Then, the structural similarity of corresponding patches is calculated from three aspects of brightness, structure and contrast. The block with the smallest value is the unit where the brain tumor is located. In the window sliding process, if the step size is set too small, the accuracy of the obtained tumor unit is improved, but the time complexity increases. If the step size is set too large, the detailed features are lost and the error increased. In order to strike a balance between computational efficiency and resulting performance, we took the stride size to 50.

### 2.3. Refined Segmentation

Chan and Vese proposed the Chan-Vese (CV) model by the regional feature information of the image. The specific energy function is:

$$\begin{array}{c} F\left(\phi ,c_{1},c_{2}\right)=\mu \int_{\Omega }H(\phi (x,y))dxdy+\lambda_{1}\int_{\Omega }{\left|I(x,y)-c_{1}\right|}^{2}H(\phi (x,y))dxdy\\ +\lambda_{2}\int_{\Omega }{\left|I(x,y)-c_{2}\right|}^{2}(1-H(\phi (x,y)))dxdy \end{array}$$

where, $c_{1}=\frac{\int_{\Omega }I(x,y)H(\varphi (x,y))dxdy}{\int_{\Omega }H(\varphi (x,y))dxdy},c_{2}=\frac{\int_{\Omega }I(x,y)\left[1-H(\varphi (x,y))\right]dxdy}{\int_{\Omega }\left[1-H(\varphi (x,y))\right]dxdy}$, *c*_1_ and *c*_2_ are gray mean of the target area and the background area: $H(\phi )=\{\begin{array}{cc} 1 & \phi \geq 0\\ 0 & \phi <0 \end{array}$. 

**Figure 6:**
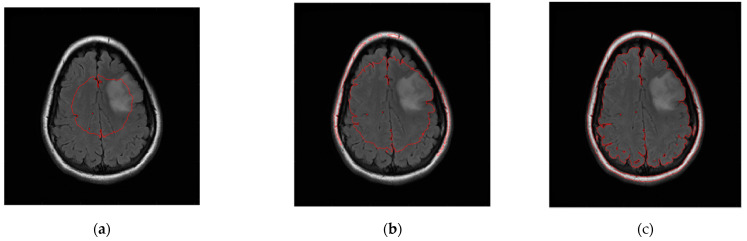
Brain tumor segment by CV model under different numbers of iterations. (**a**) 50 iterations. (**b**) 300 iterations. (**c**) 1000 iterations.

The level set initialization is required to segment the image by the CV model, and the average gray values *c*_1_ and *c*_2_ of the foreground and background are initially estimated according to the initialized level set. Then, each point on the level set is updated through the evolution equation. If the gray value of the current point is close to the gray average value of the foreground, the value of the corresponding level set of this point increases, otherwise it decreases. In CT and MRI images, the grayscale difference between brain tumors and background images is very small, and the boundaries are blurred. It is difficult to achieve satisfactory segmentation when directly applying the CV model to cross-sectional images for brain tumor detection. As shown in Figure 6, the CV model fails to converge to the location of the brain tumor.

A PDE can be obtained after variation of the CV model, and the Hermite interval Shannon-cosine wavelet can better handle the boundary conditions for the solution of the PDE. When we solve PDE through multi-scale Shannon-cosine wavelet, the wavelet coefficients can distinguish and judge the boundary of the tumor region to achieve accurate segmentation. 

Variation of the CV model yields the following partial differential equation:

$$\frac{\partial \phi }{\partial t}=\delta_{\varepsilon }(\phi )\left[\mu \mathrm{div}\left(\frac{\nabla \phi }{\left|\nabla \phi \right|}\right)+\lambda_{1}{\left|I_{0}-c_{1}\right|}^{2}-\lambda_{2}{\left|I_{0}-c_{2}\right|}^{2}\right]$$

$$\delta_{\varepsilon }=\frac{\varepsilon }{\pi \left(\varepsilon^{2}+\phi^{2}\right)}$$

where $\mathrm{div}\left(\frac{\nabla \phi }{\left|\nabla \phi \right|}\right)$ is the curvature of the contour curve, div is divergence operator.

The level set function can be expressed as:

(9) $$\begin{array}{c} \phi^{J(m,n)}(x,y,t)=\sum_{k_{01}=0}^{1}\sum_{k_{02}=0}^{1}\phi \left(x_{k_{01}}^{0},y_{k_{02}}^{0}\right)w_{k_{01},k_{02}}^{0(m,n)}(x,y)+\sum_{j=0}^{J-1}\sum_{k_{11}=0}^{2^{j}-1}\sum_{k_{12}=0}^{2^{j}-1}[\alpha_{j,k_{11},k_{12}}^{1}(t)w_{2k_{11}+1,2k_{12}}^{j+1(m,n)}(x,y)\\ +\alpha_{j,k_{11},k_{12}}^{2}(t)w_{2k_{11},2k_{12}+1}^{j+1(m,n)}(x,y)+\alpha_{j,k_{11},k_{12}}^{3}(t)w_{2k_{11}+1,2k_{12}+1}^{j+1(m,n)}(x,y)] \end{array}$$

According to the interpolation wavelet transform theory, we can obtain the wavelet coefficients: $\alpha_{j,k_{1},k_{2}}^{1}(t_{n+1})$, $\alpha_{j,k_{1},k_{2}}^{2}(t_{n+1})$, $\alpha_{j,k_{1},k_{2}}^{3}(t_{n+1})$:

$$\begin{array}{l} \alpha_{j,k_{11},k_{12}}^{1}(t_{n+1})=\phi^{J(m,n)}(x_{2k_{1}+1}^{j+1},y_{2k_{2}}^{j+1})-I_{j}\phi^{J(m,n)}(x_{2k_{1}+1}^{j+1},y_{2k_{2}}^{j+1})\\ =\phi^{J(m,n)}(x_{2k_{1}+1}^{j+1},y_{2k_{2}}^{j+1})-[\sum_{k_{01}=0}^{1}\sum_{k_{02}=0}^{1}\phi \left(x_{k_{01}}^{0},y_{k_{02}}^{0}\right)w_{k_{01},k_{02}}^{0}(x_{2k_{1}+1}^{j+1},y_{2k_{2}}^{j+1})\\ +\sum_{j=0}^{j-1}\sum_{k_{11}=0}^{2^{j_{1}}}\sum_{k_{12}=0}^{2^{j_{1}}}(\alpha_{j,k_{11},k_{12}}^{1}(t)w_{2k_{11}+1,2k_{12}}^{j+1(m,n)}\cdot (x_{2k_{1}+1}^{j+1},y_{2k_{2}}^{j+1})+\alpha_{j,k_{11},k_{12}}^{2}(t)w_{2k_{11},2k_{12}+1}^{j_{1}+1}\cdot (x_{2k_{1}+1}^{j+1},y_{2k_{2}}^{j+1})\\ +\alpha_{j_{1},k_{11},k_{12}}^{3}w_{2k_{11}+,2k_{12}+1}^{j_{1}+1}\cdot (x_{2k_{1}+1}^{j+1},y_{2k_{2}}^{j+1}))]\\ \alpha_{j,k_{11},k_{12}}^{2}(t_{n+1})=\phi (x_{2k_{1}}^{j+1},y_{2k_{2}+1}^{j+1})-I_{j}\phi (x_{2k_{1}}^{j+1},y_{2k_{2}+1}^{j+1})\\ =\phi (x_{2k_{1}}^{j+1},y_{2k_{2}+1}^{j+1})-[\sum_{k_{01}=0}^{1}\sum_{k_{02}=0}^{1}\phi \left(x_{k_{01}}^{0},y_{k_{02}}^{0}\right)w_{k_{01},k_{02}}^{0}(x_{2k_{1}}^{j+1},y_{2k_{2}+1}^{j+1})\\ +\sum_{j=0}^{j-1}\sum_{k_{11}=0}^{2^{j_{1}}}\sum_{k_{12}=0}^{2^{j_{1}}}(\alpha_{j_{1},k_{11},k_{12}}^{1}w_{2k_{11}+1,2k_{12}}^{j_{1}+1}\cdot (x_{2k_{1}}^{j+1},y_{2k_{2}+1}^{j+1})+\alpha_{j,k_{11},k_{12}}^{2}w_{2k_{11},2k_{12}+1}^{j_{1}+1}\cdot (x_{2k_{1}}^{j+1},y_{2k_{2}+1}^{j_{1}+1})\\ +\alpha_{j_{1},k_{11},k_{12}}^{3}w_{2k_{11}+1,2k_{12}+1}^{j_{1}+1}\cdot (x_{2k_{1}}^{j+1},y_{2k_{2}+1}^{j+1}))]\\ \alpha_{j,k_{1},k_{2}}^{3}=\phi (x_{2k_{1}+1}^{j+1},y_{2k_{2}+1}^{j+1})-I_{j}\phi (x_{2k_{1}+1}^{j+1},y_{2k_{2}+1}^{j+1})\\ =\phi (x_{2k_{1}+1}^{j+1},y_{2k_{2}+1}^{j+1})-[\sum_{k_{01}=0}^{1}\sum_{k_{02}=0}^{1}\phi \left(x_{k_{01}}^{0},y_{k_{02}}^{0}\right)w_{k_{01},k_{02}}^{0}(x_{2k_{1}}^{j+1},y_{2k_{2}+1}^{j+1})\\ +\sum_{j=0}^{j-1}\sum_{k_{11}=0}^{2^{j_{1}}}\sum_{k_{12}=0}^{2^{j_{1}}}(\alpha_{j_{1},k_{11},k_{12}}^{1}w_{2k_{11}+1,2k_{12}}^{j_{1}+1}\cdot (x_{2k_{1}}^{j+1},y_{2k_{2}+1}^{j+1})+\alpha_{j,k_{11},k_{12}}^{2}w_{2k_{11},2k_{12}+1}^{j_{1}+1}\cdot (x_{2k_{1}}^{j+1},y_{2k_{2}+1}^{j_{1}+1})\\ +\alpha_{j_{1},k_{11},k_{12}}^{3}w_{2k_{11}+1,2k_{12}+1}^{j_{1}+1}\cdot (x_{2k_{1}}^{j+1},y_{2k_{2}+1}^{j+1}))] \end{array}$$

where *w_k,j_* is the interval interpolation basis functions, and $\phi_{n}^{j}$ is Shannon-Cosine wavelet [29]:

$$w_{k,j}\left(x\right)=\{\begin{array}{ll} \phi \left(2^{j}x-0\right)+\sum_{n=1}^{N}{\left(1-\frac{n}{N}\right)}^{2}\left(1+n+\frac{2n}{N}\right)\phi \left(2^{j}x+n\right), & k=0\\ \phi \left(2^{j}x-1\right)+\sum_{n=1}^{N}n{\left(1-\frac{n}{N}\right)}^{2}\phi \left(2^{j}x+n\right), & k=1\\ \phi (2^{j}x-k), & k=2,3,\cdots ,2^{j}-2\\ \phi \left(2^{j}x-2^{j}+1\right)+\sum_{n=1}^{N}n{\left(1-\frac{n}{N}\right)}^{2}\phi \left(2^{j}x-2^{j}-n\right), & k=2^{j}-1\\ \phi \left(2^{j}x-2^{j}\right)+\sum_{n=1}^{N}{\left(1-\frac{n}{N}\right)}^{2}\left(1+n+\frac{2n}{N}\right)\phi \left(2^{j}x-2^{j}-n\right), & k=2^{j} \end{array}\;\begin{array}{l} \phi_{n}^{j}\left(x\right)=\phi \left(x-x_{n}^{j}\right)\\ =\frac{\sin\frac{\pi }{\Delta_{j}}\left(x-x_{n}^{j}\right)}{\frac{\pi }{\Delta_{j}}\left(x-x_{n}^{j}\right)}\sum_{n=0}^{m}\left(a_{n}\cos\left(\frac{2n\pi }{N}\left(x-x_{n}^{j}\right)\right)\right)\cdot \left[\chi \left(\left(x-x_{n}^{j}\right)+\frac{N}{2}\right)-\chi \left(\left(x-x_{n}^{j}\right)-\frac{N}{2}\right)\right] \end{array}$$

We defined *ϕ* and its derivative is:

(10) $$\frac{\partial \phi }{\partial t}=F\left(t,x,y,\phi ,\frac{\partial \phi }{\partial x},\frac{\partial \phi }{\partial y},\frac{\partial^{2}\phi }{\partial x^{2}},\frac{\partial^{2}\phi }{\partial x\partial y},\frac{\partial^{2}\phi }{\partial y^{2}}\right)$$

$$\begin{array}{l} \frac{d\phi^{J}(x,y,t)}{dt}=F[t,x,y,\phi^{J}(x,y,t),\phi^{J(1,0)}(x,y,t),\phi^{J(0,1)}(x,y,t),\\ \phi^{J(2,0)}(x,y,t),\phi^{J(1,1)}(x,y,t),\phi^{J(0,2)}(x,y,t)] \end{array}$$

$$F[t_{n},x,y,\phi^{J}(x,y,t_{n}),\phi^{J(1,0)}(x,y,t_{n}),\phi^{J(0,1)}(x,y,t_{n}),\phi^{J(2,0)}(x,y,t_{n}),\phi^{J(1,1)}(x,y,t_{n}),\phi^{J(0,2)}(x,y,t_{n})]$$

is denoted by *F_n_*.

We can construct a linear homotopy model:

(11) $$\phi^{J}(x,y,t)=(1-\varepsilon )F_{n}+\varepsilon F_{n+1}$$

where *ε*(*t*) is the homotopy parameter. $\varepsilon (t)=\frac{t-t_{n}}{t_{n+1}-t_{n}}\;t\in \left[t_{n},t_{n+1}\right],\varepsilon \in [0,1]$.

According to perturbation theory, the solution of Equation (11) is:

$$\phi^{J}(x,y,t)=\phi_{0}^{J}(x,y,t)+\varepsilon \phi_{1}^{J}(x,y,t)+\varepsilon^{2}\phi_{2}^{J}(x,y,t)+\cdots$$

Substituting Equation (9) into Equation (10):

$$\begin{array}{l} \varepsilon^{0}:\phi_{0}^{J}=F_{n}\\ \varepsilon^{1}:\phi_{1}^{J}=F_{n+1}-F_{n} \end{array}$$

Substituting the wavelet transform coefficient into Equation (9), we have:

$$\begin{array}{l} \phi^{J}(x,y,t_{n+1})=\phi_{0}^{J}(x,y,t_{n})+\frac{\Delta t}{2}[F(t_{n},x,y,\phi^{J}(x,y,t_{n}),\phi_{}^{J(1,0)}(x,y,t_{n}),\phi_{}^{J(0,1)}(x,y,t_{n}),\\ \;\phi_{}^{J(2,0)}(x,y,t_{n}),\phi^{J(1,1)}(x,y,t),\phi^{J(0,2)}(x,y,t))]+F(t_{n+1},x,y,\phi_{0}^{J}(x,y,t_{n+1}),\\ \;\phi_{0}^{J(1,0)}(x,y,t_{n+1}),\phi_{0}^{J(0,1)}(x,y,t_{n+1}),\phi_{0}^{J(2,0)}(x,y,t_{n+1}),\phi_{0}^{J(1,1)}(x,y,t_{n+1}),\phi_{0}^{J(0,2)}(x,y,t_{n+1})) \end{array}$$

**Figure 7:**
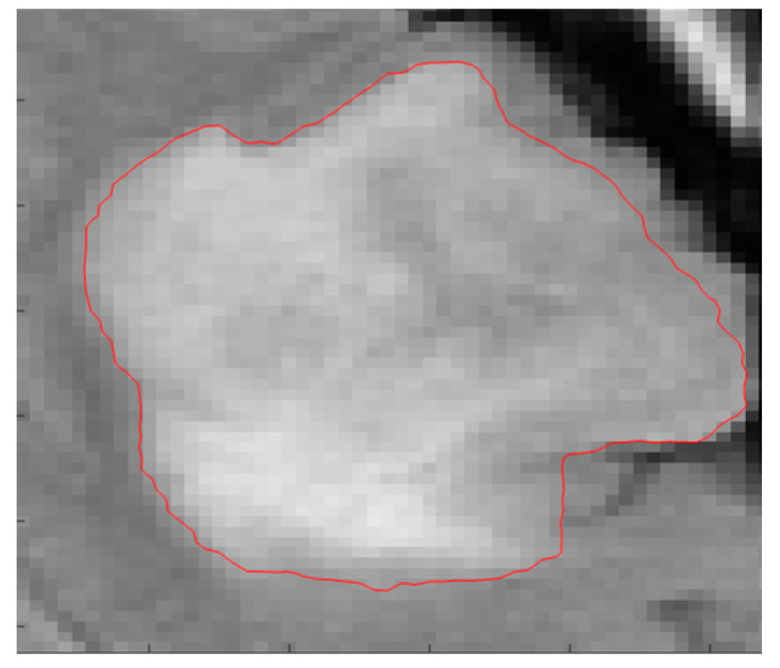
The improved CV model detection.

After locating the unit area where the tumor is located in Section 2.2, we can segment the tumor tissue through the improved CV model, so that the tumor tissue can be accurately detected and segmented, as shown in Figure 7.

## 3. Results

### 3.1. Performance Evaluation Metrics

We evaluated the segmentation performance by Accuracy, JSC, DSC, which are described as follows:

(12) $$\mathrm{Accuracy}=\frac{\mathrm{TP}+\mathrm{TN}}{\mathrm{TP}+\mathrm{TN}+\mathrm{FP}+\mathrm{FN}}$$

(13) $$\mathrm{JSC}=\frac{\mathrm{TP}}{\mathrm{TP}+\mathrm{FP}+\mathrm{FN}}$$

(14) $$\mathrm{DSC}=\frac{2\mathrm{TP}}{\mathrm{FP}+2\mathrm{TP}+\mathrm{FN}}$$

where True Positive (TP): correct identification, and False Negative (FN): incorrect rejection. True Negative (TN): correct rejection and False Positive (FP): incorrect identification, as shown in Table 1.

In addition, paired *t*-test was performed on the proposed model and other models in test 6 for comparison with respect to these evaluation metrics. *p*-value of <0.05 was considered statistically significant.

### 3.2. Comparison of Segmentation Results with Different Methods

**Figure 8:**
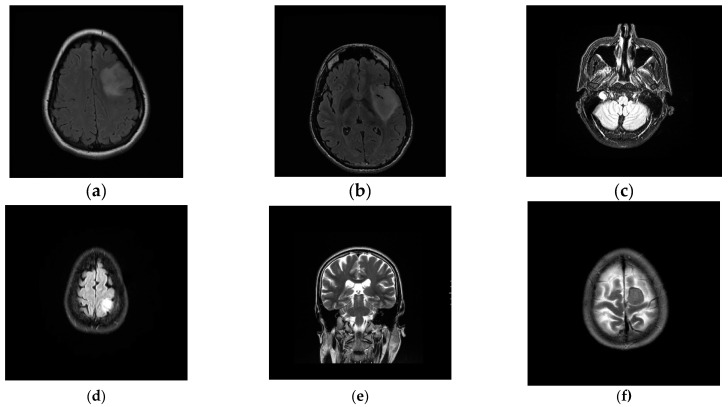
Examples of brain tumor images. (**a**) Test1. (**b**) Test2. (**c**) Test3. (**d**) Test4. (**e**) Test5. (**f**) Test6.

**Figure 9:**
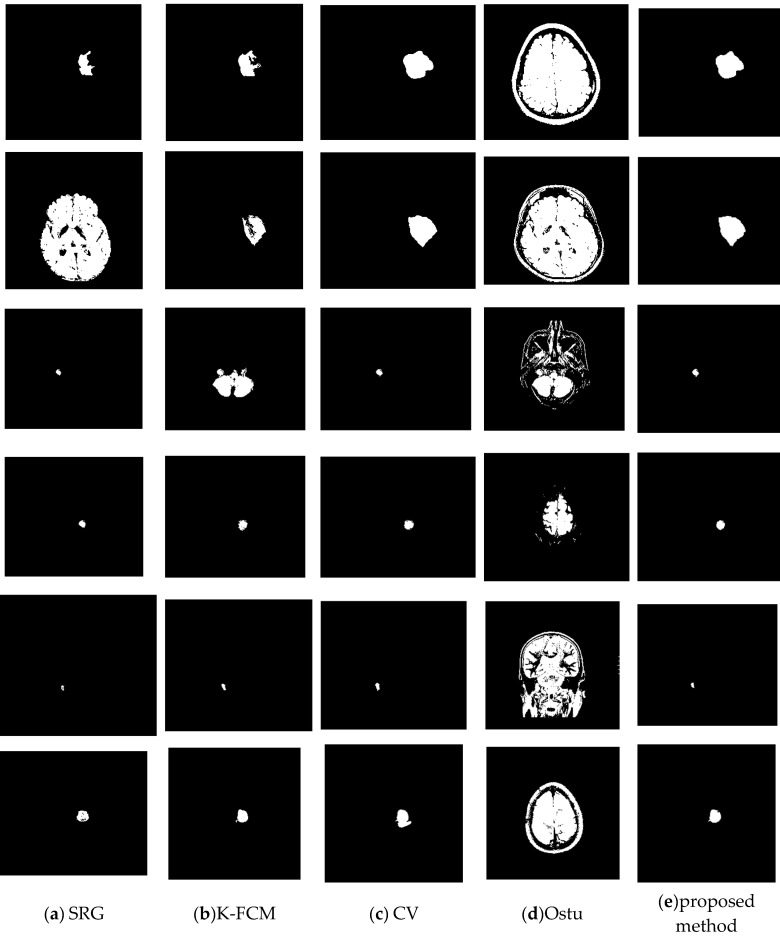
Comparison of the proposed method with others. (**a**) SRG. (**b**) K-FCF. (**c**) CV. (**d**) Ostu. (**e**) The proposed method.

To verify the effectiveness of the proposed method, we randomly selected some images to test. For each brain tumor cross-sectional image, we compared the results of each algorithm with the results of manual segmentation. Figure 8 is the original image. Figure 9 shows the visualization results obtained by the proposed algorithm, CV, K-FCM [9], Ostu [30] and region growing algorithm [8] for brain tumor segmentation. The experimental results of the threshold algorithm were obtained by manually adjusting the threshold parameters several times. Except for the algorithm in this paper, the other methods could not fully achieve automated detection and segmentation. From Table 2, we can observe the quantitative results of the four detection algorithms for brain tumor. When Accuracy, JSC and DSC were higher, it indicated that the prediction accuracy of the target was higher. The proposed method outperformed other algorithms on Accuracy, JSC, and DSC. Therefore, from the comprehensive analysis in Figure 9 and Table 2, it can be seen that the algorithm in this study had high accuracy in detecting and segmenting brain tumors. The proposed method has certain competitiveness compared with other classical algorithms, and is expected to provide a reliable reference for clinical decision-making. In addition, as is shown in Table 3, there are significant differences between the proposed model and other models.

## 4. Conclusions

Owing to the variability and fuzzy boundary of brain tumor lesions, it is very challenging to develop an automated tumor detection system. We introduced Bendlet into cross-sectional images to extract the dominant features between the normal and abnormal images and realized image registration and correction. Meanwhile, the block where the brain tumor was located was assessed from three aspects of brightness, structure and contrast, which enabled driving the contour line of the improved CV model to the required boundary, even near the weak edge. The ideas presented in this work also offer a potential direction to improve detection accuracy for all types of medical diagnoses.

## Figures and Tables

**Table 1:** Brain tumors segmentation outcome.

Confusion Matrix		Real	
		Positive	Negative
**Predict**	Positive	TP	FP
	Negative	FN	TN

**Table 2:** Comparative results on six main metrics using different methods.

		Accuracy	JSC	DSC
Test1	K-FCM	0.9917	0.6542	0.7910
	CV	0.9948	0.8109	0.8956
	SRG	0.9883	0.4941	0.6614
	Ostu	0.7415	0.0810	0.1498
	Ours	0.9955	0.8298	0.9070
Test2	K-FCM	0.9944	0.7744	0.8729
	CV	0.9962	0.8653	0.9278
	SRG	0.7948	0.1018	0.1849
	Ostu	0.7363	0.0847	0.1563
	Ours	0.9972	0.8993	0.9470
Test3	K-FCM	0.9591	0.0302	0.0587
	CV	0.9996	0.7856	0.8799
	SRG	0.9997	0.8297	0.8681
	Ostu	0.9132	0.0147	0.0290
	Ours	0.9999	0.9428	0.9705
Test4	K-FCM	0.9991	0.7750	0.8732
	CV	0.9992	0.8032	0.8908
	SRG	0.9986	0.6046	0.7536
	Ostu	0.9611	0.0825	0.1524
	Ours	0.9994	0.8381	0.9119
Test5	K-FCM	0.9988	0.4384	0.6096
	CV	0.9986	0.3453	0.5134
	SRG	0.9984	0.2340	0.3792
	Ostu	0.8133	0.0109	0.0215
	Ours	0.9990	0.5322	0.6947
Test6	K-FCM	0.8995	0.9276	0.9624
	CV	0.6966	0.6449	0.7841
	SRG	0.7585	0.7793	0.8759
	Ostu	0.9374	0.0017	0.0034
	Ours	0.9995	0.9285	0.9629

**Table 3:** The Paired *t*-test for the proposed model and other models (*p*-Value).

	Accuracy	JSC	DSC
K-FCM-Ours	0.040913566	0.080455	0.10538
CV-Ours	0.17593634	0.02078	0.025113
SRG-Ours	0.080970625	0.01242	0.021317
Ostu-Ours	0.007289404	2.65 × 10^−5^	5.41 × 10^−6^

## Data Availability

Some of the data supporting the findings of this study are openly available at https://www.kaggle.com/datasets/mateuszbuda/lgg-mri-segmentation. Additional data is provided by Clinic of the Bashkir State Medical University. (accessed on 1 March 2022).
